# Supplementary material for: Are carbon emissions trading and green financial instruments synergistic? -Comprehensive quantitative research based on content analysis
Source: PLoS One. 2024 Mar 7;19(3):e0298601. doi: 10.1371/journal.pone.0298601 (PMC10919601; doi:10.1371/journal.pone.0298601)
Supplement: S1 Data — (ZIP) [file pone.0298601.s001.zip › Data/地级市Z_F工作报告.zip!/╕╩╦α/╟∞╤⌠╩╨/└┤╫╘╣1⁄2╓┌║┼í╛┬φ┐╦╩2╛▌═°í┐_2014.docx]

各位代表：

现在，我代表市人民政府，向大会作工作报告，请予审议，并请各位政协委员和列席人员提出意见。

一、坚持稳中求进，2013年政府工作取得新成效

2013年，在省委、省政府和市委的坚强领导下，我们认真贯彻党的十八大和十八届三中全会及省十二次党代会精神，全面落实习近平总书记系列重要讲话精神，准确把握“稳中求进”总基调，主攻“一区四园、一线八域”主战场，突出“十大工程”主抓手，着力调结构、转方式，破瓶颈、强基础，惠民生、促和谐，完成了市三届人大三次会议确定的目标任务。

（一）经济发展稳健向好。面对经济形势错综复杂、挑战压力巨大的特殊形势，深入研究落实一系列稳增长、调结构、促转型的有效措施，全市经济稳健增长，发展势头持续向好，质量效益同步提升，后劲活力明显增强，主要经济指标实现了历史性突破。预计全年生产总值突破600亿元，达到616.5亿元，增长16.2%；规模以上工业增加值达到333亿元，增长18.1%；固定资产投资突破1000亿元，达到1053亿元，增长40%；社会消费品零售总额突破150亿元，达到154.5亿元，增长18.1%；财政总收入突破150亿元，达到154.3亿元，增长18.7%，公共财政收入达到63.7亿元，增长20%；城镇居民人均可支配收入达到19578元，增长17.5%；农民人均纯收入突破5000元，达到5063元，增长18.8%。

（二）首位产业开发加快。通过深入调研和专家论证，能源化工首位产业的战略地位更加凸显，石油石化、煤炭生产转化“两个千亿元产业链”规划设计、开发路径和转化模式进一步明确，“绿色、循环、低碳、高效”发展理念成为全市上下的鲜明共识。石油开发“双千万”工程顺利推进，原油产量659万吨、加工量341万吨，庆阳石化600万吨炼油升级改造工程全面启动，拉开了石油炼化一体化发展的序幕。煤炭开发转化工程步伐加快，6个煤炭区块勘查有序推进，刘园子矿井投产试运行，甜水堡、核桃峪、新庄矿井建设进展顺利，正宁电厂一期工程扎实推进，50万吨合成氨、80万吨尿素和100万吨缓释高效肥项目启动实施。天然气、煤层气勘探开发取得新进展。环县百万千瓦风电项目建设加快推进。

（三）富民增收成效显著。种植业、养殖业和区域结构调整力度加大，全膜双垄沟播面积增加，粮食生产在多灾之年保持稳产增产，总产量达到158.9万吨。1县1乡3村现代农业综合示范工程取得实效，特色富民产业主导地位进一步凸显，肉羊、肉鸡等特色养殖规模、标准和效益显著提升。苹果、蔬菜等绿色高效产业品牌化、标准化建设取得新突破，庆阳苹果、庆阳香包、环县皮影列入国家地理标志保护产品目录。新一轮扶贫攻坚大幕全面拉开，贫困群众脱贫致富的门路进一步拓宽，减少贫困人口14.7万人。农民技能培训工程深入实施，培训农民32.5万人（次）。新增农民合作社976个，建成家庭农场19个，辐射带动农民15.7万户。“万村千乡”市场工程信息化、县乡农贸市场标准化改造年度任务全面完成。汽车、通讯、文化、旅游等消费势头强劲，文化产业增加值增长58%，第三产业吸纳就业、富民增收的能力不断增强。

（四）基础条件持续改善。严格落实重点项目“三个一”包抓责任制和盯守办理制，全年实施500万元以上项目2799个，实现了投资增长与结构优化双突破。庆阳机场新增了北京航线；西平铁路全线贯通，结束了我市境内不通铁路的历史；银西铁路获得国家发改委批复立项；西雷高速建成通车，甜罗高速列入国家高速路网规划；宁长、西镇、新南3条二级公路建成通车；新修农村公路124条1040公里。修建各类水利工程3532处，解决了26万人的安全饮水困难。城乡电网改造步伐加快，2个110千伏输变电工程建成投运。市区路网、供水供热、排洪排污、公园绿地等工程建设协调推进，7个县城和30个重点小城镇建设成效显著，城镇化率提高3个百分点。百万亩土地整治、雨洪集蓄生态保塬、宜林荒山苗林产业培育工程建设全面展开，新修梯田47.3万亩，造林42.4万亩，创建省级生态乡镇47个、生态村26个。

（五）改革开放不断深化。农村集体土地确权登记和集体林权制度配套改革进展顺利。创新乡村道路管理机制，农村交通保障能力显著提升。取消、下放、调整行政审批事项133项。市公共资源交易中心启动运行。食品药品、工商管理、质量技术监督管理体制初步调整理顺。“营改增”工作分类推进。引进商业银行2家，成立小额贷款公司19家。能源化工集团实现了当年组建、当年运营、当年盈利，交通、水务集团组建运营。市经济技术开发区规划通过省级评审，承接产业转移项目75个。对外开放不断扩大，成功举办了中国（庆阳）循环经济•绿色发展战略论坛、2013中国•庆阳农耕文化节暨第24届中国西部商品交易会。招商引资到位资金451.4亿元，增长68.3%，是招商成效最大、到位资金最多的一年。

（六）社会事业全面进步。省列24件、市列10件为民办实事项目落实到位，完成投资39.3亿元。教育质量稳步提升，高考进线率较上年提高1.43个百分点，教育布局结构不断优化，改造薄弱学校53所，新建改扩建幼儿园73所，为全市395所农村小学建起了小伙房。科技成果转化运用水平提高，引进推广新技术、新产品、新品种130项，实施科技合作项目30个。文化事业快速发展，广播电视信号覆盖面进一步扩大，群众文化体育活动蓬勃发展。医疗卫生服务体系不断健全，35个乡镇医疗服务中心和113个村卫生室建成投用，新农合报销医疗费7亿元，受益群众218万人（次）。计划生育工作得到加强，完成了年度人口控制目标。就业“双导”工程和“百企千岗”创业促就业活动富有成效，招录3145名高校毕业生到基层就业，新增城镇就业4.35万人，输转城乡富余劳动力60.5万人。社会保障覆盖面持续扩大，城乡低保标准分别提高10%和13.5%。建成保障性住房5556套，实施农村危房（窑）改造1.56万户。暴洪灾后重建任务基本完成。平安和谐庆阳创建工程成效良好，矛盾纠纷排查调处和信访工作扎实有效，社会大局和谐稳定。道路交通、煤矿、非煤矿山、食品药品、校园、消防、生产等重点领域安全监管力度加大。少数民族乡村发展进一步加快，夏河藏区援建任务落实到位。审计、统计、国防动员、双拥、人防、地震、气象、档案、老龄、残疾人、外事侨务等工作取得了新进步。

（七）政府建设扎实推进。认真执行市委的决策部署，“一区四园”建设步伐加快，管理体制不断健全，“一线八域”发展成效明显，“十大工程”建设进展良好。自觉接受人大依法监督和政协民主监督，积极支持人大代表、政协委员开展视察、调研活动，办理人大代表建议84件、政协委员提案315件。加强与民主党派和群团组织的联系，广泛听取社会各界对政府工作的意见建议，科学化、民主化决策机制进一步完善。认真贯彻中央“八项规定”、国务院“约法三章”、省委“双十条规定”和市委“实施细则”，制定出台了7项财政管理制度，全市“三公”和会议经费支出下降32.7%。“双联”行动和“效能风暴”行动深入开展，干部作风明显改进，政府系统廉政建设取得新成效。

在肯定成绩的同时，我们也清醒地看到，政府工作还存在一定差距和不少困难。任务完成方面，受国家宏观政策调整所限，谋划的一些重大交通、能源等项目争取审批难度大、周期长，计划开工的银西铁路、甜罗高速和3个水库未能实现预期，原油生产、加工未完成市列计划。经济结构方面，能源化工产业关联度、融合度不强，地方工业弱小；农业种养结构不合理，农民收入持续增长能力不强；第三产业增长慢，拉动需求不足，市场消费不旺。群众生活方面，一些贫困乡村道路、饮水、住房、用电等问题还十分突出，部分群众生活困难，公共服务、社会保障、社会治理的能力和水平需要进一步提升。工作落实方面，个别干部工作作风不扎实，一些工作任务落实不到位，“四风”问题在一些行业和领域还有不同程度的表现。这些困难和问题，需要我们切实加以克服和解决。

一年来的政府工作实践，更加深化了全市上下推动科学发展、加快转型升级、促进富民兴市的共识。我们深深地感到：只有把培育壮大首位产业作为第一抓手，才能实现建设能源新都的战略目标；只有把“3341”项目工程作为第一引擎，才能不断做大总量、调优结构、转变方式；只有把深化改革作为第一动力，才能解放和发展生产力，全面释放改革“红利”；只有把保障和改善民生作为第一任务，才能让老百姓过上更有保障、更加幸福的生活；只有把强化执行作为第一保障，才能把决策变成实践，把目标变成现实。

各位代表，成绩来之不易，经验弥足珍贵。这些成绩的取得，是省委、省政府正确领导、亲切关怀的结果，是市委统揽全局、科学决策的结果，是市人大、市政协倾力支持、有效监督的结果，是全市广大干部群众团结奋斗、辛勤努力的结果。在此，我谨代表市人民政府，向各位代表和政协委员，向各民主党派、工商联、人民团体和无党派人士，向离退休老同志，向驻庆人民解放军、武警官兵和中央、省属单位，向奋战在全市各条战线上的劳动者，向所有关心、支持庆阳发展的社会各界人士，致以崇高的敬意和衷心的感谢！

二、全面深化改革，努力开创2014年发展新局面

2014年，是深入贯彻落实党的十八届三中全会精神、全面深化改革的开局之年，是加快全面建成小康社会、实现“十二五”发展目标的关键一年。从有利因素看，国家创新宏观调控方式，明确支持中西部地区加快基础设施建设，为我们破解基础瓶颈、推动转型升级提供了难得机遇；省委、省政府确定的三大战略平台进入了国家层面，我省打造丝绸之路经济带甘肃黄金段，我市被国务院确定为成长型资源城市和天然气后备基地，为加快发展提供了有利条件；全市人民思富求变愿望强烈，为富民兴市提供了强劲动力。从面临挑战看，目前世界经济复苏缓慢，国内经济不确定因素仍然很多；石油、煤炭等商品价格波动对我市经济发展影响不可忽视，以能源资源为主导的支柱产业发展还存在不少挑战；传统的能源开发方式与绿色、低碳发展的要求还不相适应。我们只有把困难问题剖析得更深透一些，把面临的复杂形势估计得更充分一些，才能化困难为动力，变挑战为机遇，在新的一年里谋求各项工作的更大创新和突破。

按照中央、省上和市委的总体部署，统筹考虑国际国内形势和我市发展的阶段性特征，准确把握政策机遇和风险挑战，今年政府工作的总体要求是：全面贯彻落实党的十八大和十八届三中全会精神，全面贯彻落实中央和全省经济工作会议、城镇化工作会议精神，牢牢把握稳中求进和改革创新这一核心，牢牢把握提高质量和效益这一中心，以“一区四园、一线八域”为主战场，以“十大工程”为主抓手，更加注重把握稳增长、调结构的重点问题，更加注重推进转方式、增活力的重点改革，更加注重强化促发展、惠民生的重点工作，加快建设幸福美好新庆阳。

预期目标是：全市生产总值增长13%，固定资产投资增长28%，公共财政收入增长13%，社会消费品零售总额增长15%，城镇居民可支配收入增长13%，农民人均纯收入增长16%。

实现上述目标，做好今年政府工作，最关键的就是把稳中求进、改革创新这一核心要求落到实处，做到“稳”有定力、“进”有效果，“改”有秩序、“创”有作为。

稳中求进，就是切实解决好“五个重点问题”。一是预期性目标的合理增速。基于对我国发展大势的把握，基于省上对我市目标定位的衡量，基于对我市发展基础和转型升级要求的考虑，科学把握发展速度和质量的平衡点、经济发展和民生改善的契合点，使各项预期目标增速在合理区间运行，保持“稳”这一前提和基础，突出“进”这一行动和目标。二是约束性指标的严格控制。牢固树立“绿色、循环、低碳、高效”的发展理念，遵循发展规律、经济规律、自然规律，确保单位GDP能耗和主要污染物排放等指标控制在省列目标之内，促进发展由“黑色印象”向“绿色主题”转变。三是扶贫与小康的统筹推进。既量力而行，盯住小康社会统计监测指标，倒逼各项工作任务落实，确保在全面小康建设中不掉队；又尽力而为，全力打好扶贫攻坚战，确保贫困群众收入增长20%左右。四是政府债务的风险防控。把防控和化解政府债务风险作为一项重要任务和硬性考核指标，开展政府债务审计清查工作，落实偿债主体责任，积极稳妥地化解政府债务，确保逾期债务偿还率达到10%。五是民生保障的制度设计。按照“普惠制、全覆盖、均等化、可持续”的原则，统筹谋划保障和改善民生工作，使“碎片化”的民生政策系统化；把当前、近期和长远的民生工程建设有机统一起来，把民生工程与经济工作同安排、同落实、同考核，集中财力办大事、解难事，使民生工作常态化。

改革创新，就是按照中央、省上和市委关于全面深化改革的要求，正确、稳步、有序、协调推进“六项重点改革”：一是深化行政管理体制改革。坚持民意为先、舍利为公，认真做好行政审批事项的“接、放、管”工作，贯彻落实《关于市县政府职能转变和机构改革的指导意见》，积极推进卫生和计划生育、工商、质监等行政机构改革，稳步推进事业单位分类改革，努力使行政效率更高、服务效能更好、发展环境更优。二是发展混合所有制经济。研究制定加快混合所有制经济发展的意见，推动地方企业融入能源开发转化的大型央企和知名民企，促进各种所有制经济共同发展、共同繁荣。三是推进农村综合改革。继续深化农村经营体制改革和配套改革，探索建立农民增收、农村公共服务供给、农业人口转移、统筹城乡规划和建设、经营性集体建设用地入市等体制机制，使农民资产“动”起来、农村产业“活”起来，持续增加农民收入。四是完善地方金融服务体系。建立银政、银企合作长效机制，促进地方经济与金融发展深度融合。实施“引行入庆”行动，抓好国开行金融支持产业扶贫开发试点工作。发展和规范村镇银行、小额贷款公司和农村资金互助合作组织。改善信用环境，维护金融安全。五是深化资源市场改革。坚持“公平、开放、透明”的原则，争取建立不分所有制、不分城乡、统一开放的生产要素市场准入制度，依据市场规则、市场价格、市场竞争配置资源，把优势资源配置给优势企业，开发优势产业，实现效益最大化。六是完善生态文明制度建设。坚持“谁受益、谁补偿”的原则，争取建立生态环境保护补偿治理机制。探索推进环境监测执法改革和国有林场改革，加快编制生态文明建设规划，划定生态保护红线，加大封山禁牧力度。创新生态建设投入机制，动员市内外个人、团体和企业积极参与宜林荒山苗林产业开发，明晰产权，放活经营权，每年造林100万亩，力争七年“再造一个子午岭”。

三、培育壮大能源化工首位产业，推动经济转型升级

围绕优势资源开发，做大做强能源化工首位产业，在提升能源资源就地消化能力上下功夫，在破解煤、电外送问题上求突破；突出扩大内需，加快现代服务业发展，在调结构、转方式中不断提升发展质量和效益，实现经济转型升级。

着力打造石油石化千亿元产业链。支持油田企业扩能上产，确保原油产量完成750万吨；加快庆阳石化600万吨炼油升级改造项目建设，确保加工量完成350万吨。积极开展后续石化项目前期工作，促进石油资源由原料输出型向转化增值型转变。加大天然气勘探开发力度，推进探井评价试采，抓好日处理90万立方米天然气综合利用项目建设，完成“气化庆阳”年度任务。

积极培育煤炭生产转化千亿元产业链。强化投产矿井的生产管理，力争出煤40万吨以上。加快3个在建矿井和正宁电厂一期建设进度，开工建设1个矿井，做好3个矿井、环县电厂和西峰、长庆桥热电联产项目前期工作。探索建立地企联合循环经济示范区，建设地方自备电厂，吸引现代高载能企业入驻开发。通过煤制肥、煤制气、煤制聚烯烃、煤制油、煤制兰炭等5条路径延长产业链，跟踪推进50万吨合成氨、80万吨尿素和100万吨缓释高效肥项目，支持晋煤、中煤天大能源公司等加快煤转化、煤层气开发、煤机制造项目建设，培育以煤炭开采为基础、煤转电为支撑、煤化工为主导、煤冶材为补充的千亿元煤电化产业集群。加快环县百万千瓦风电场建设，启动实施华池10万千瓦风电项目。

加快第三产业转型升级。着眼建立统一开放、竞争有序的市场体系，抓好市区10大专业市场建设，加快长庆桥煤炭物流园、西峰空港物流园、上海红星美凯龙大型商业区、“万村千乡”市场工程等流通网络建设。规范市场秩序，强化水、电、气、物业等经营服务管理。抢抓国家支持现代服务业发展的机遇，运用连锁经营、物流配送、区域代理等现代经营方式，改造提升传统服务业；积极培育家政服务、信息咨询、融资租赁、法律服务、电子商务等新兴服务业，加快“智慧城市”建设，促进服务业快速发展。

推进深度融合发展。加强与大型央企和知名民企的合作开发，积极探索地方与能源开发企业融合发展新机制，充分发挥能源化工集团公司龙头带动作用，主动承接油煤气开发配套服务工程，以资源兴产业，以服务换合作，促进资源开发、企业发展、百姓富裕有机统一。抢抓省上建设华夏文明传承创新区、陇东南中医药养生保健旅游创新区的机遇，以民俗庆阳、红色南梁、周祖圣地、岐黄故里“四大品牌”为引领，高层次编制全市大景区规划，多元化开发文化旅游产业。建设庆阳民俗文化产业园、山城堡战役纪念园、陕甘红军纪念园、正宁子午岭人文生态景观带等景区。提升周祖农耕文化产业园、岐黄中医药文化生态园、南梁红色小镇等文化产业聚集区的辐射带动能力，促进文化旅游融合发展。

放手发展非公有制经济。全面落实非公有制经济发展的各项扶持政策，研究制定非公有制经济进入特许经营领域的具体办法，在投资核准、融资服务、财税政策、土地使用、经济技术合作等方面，不分企业大小、不管市内市外、不论所有制形式，都享受同等待遇和服务。坚持扶持地方民企与引进知名民企相结合，深入推进“民企陇上行”活动，积极培育多元发展、多点支撑的新兴产业，提高非公经济的比重。

建设“一区四园”战略平台。积极创建国家级经济技术开发区，打造石油石化、煤电化冶材、装备制造、现代物流和文化创意产业聚集区，为建设能源新都搭建战略平台。通过市场化运作方式，拓宽筹融资渠道，加快园区建设，提升承载能力，吸引企业入驻。

四、实施项目带动战略，积极稳妥推进新型城镇化

准确把握国家支持全局性、基础性、战略性重大项目的投资导向，坚持重大基础项目、特色产业项目、民生保障项目齐抓共进，市区、城镇、农村和规划、建设、管理“两个一体化”协调推进，以重大项目建设和新型城镇化助推转型升级、富民兴市。

深入实施“3341”项目工程，夯实发展基础。严格落实项目工作“五项制度”，严把工程质量关，充分发挥项目效益。围绕提高交通支撑能力，力争银西铁路、甜罗高速和柔太红色旅游路开工，加快西合二级公路建设，确保庆阳机场航站楼建成投用。围绕提升水资源保障能力，坚持“四水齐抓”，开工建设小盘河、葫芦河、巴家咀3处水资源保障工程，做好马莲河水库、甜水堡调蓄水库和环县苦咸水淡化项目前期工作，新增有效灌溉面积3.3万亩、节水灌溉面积3万亩。围绕增强电力供给能力，完成3个110千伏和环县330千伏输变电工程建设，持续完善城乡电网。围绕提升生态承载能力，加快实施百万亩土地整治、董志塬水土保持治理、农村面源污染和荒漠化防治等工程，建设美丽乡村，创建生态市。坚持资源开发与环境保护同步推进，抓好重点行业和区域污染治理，强化大气污染防治和饮用水源地保护。

扩大开放开发，拓宽发展空间平台。积极融入新丝绸之路经济带和西咸经济圈，加强与中亚、西亚等国家和地区的合作交流，推动优质中药材、民俗文化产品和绿色农畜产品等方面的贸易往来。主动承接装备制造、高新技术和现代服务业等产业转移。积极为企业走出去牵线搭桥、提供信息，大力发展外向型经济，打造一批体现庆阳特色的贸易品牌，提高经济发展外向度和融合力，确保出口创汇增长10%。灵活运用“五个招商”模式，紧盯世界500强、国内500强企业，开展“引强入庆”行动，确保招商引资到位资金增长30%以上。持续优化“三心环境”，创新管理，强化服务，把好项目规划、功能、结构、环保和投资“五个关口”，力争一批重大项目落地实施。

推进新型城镇化，构建城乡一体化发展格局。按照中央和全省城镇化工作会议的新要求，启动新型城镇化规划编制工作，科学合理确定城镇发展定位、规模层次、空间布局和开发强度，构建以市区、特色县城、重点小城镇、工业集中区为支撑的城镇化发展体系。创新土地管理、使用机制，守住耕地“红线”，促进城镇建设用地集约、节约利用。有序放开市、县落户限制，促进城镇常住农业人口优先实现市民化。把推进城镇化和育产业、促就业紧密结合，打造宜居、宜业的特色城镇。在“长环线”选择一批重点城镇开展统筹公共服务、社会保障和新农村建设试点。加快市县旧城改造、新区开发和基础功能配套建设，实施各类城镇建设项目292项，力争城镇化率提高2个百分点。改革市区公共交通管理体制，提升保障能力，方便市民出行。持续推进文明城市创建活动，提升市民素质。

五、打好扶贫攻坚战，加快全面小康社会建设

我市农村人口多、贫困程度深、攻坚难度大。加快扶贫攻坚，与全国、全省同步全面建成小康社会，建设幸福美好新庆阳，是我市发展的根本大局，是政府的历史使命。坚持以扶贫攻坚、全面小康统揽“三农”工作全局，通过“三年攻坚、一年完善”，使农业基础稳固、农村和谐稳定、农民安居乐业。重点打好“两大攻坚战”，突出抓好“一项培训工程”。

打好以交通为牵引的基础设施攻坚战。坚持基础优先取向，以行政村为单元，深入实施农村“公路通畅、用电质量提高、饮水安全、易地扶贫搬迁”四大工程，下功夫帮助贫困乡村“换穷貌”。从今年开始，总投入资金40亿元，实施“交通扶贫三年大决战”行动，为635个村新修通村公路6268公里，实现所有建制村通油路（水泥路）的目标。加强乡村道路管护，提高通畅能力。在北部山区和南部原边咀梢，实施“一户两场四窖”和小电井工程；在中南部原区、沿河川台区，实施“千吨万人集中供水”工程，提高农村饮水安全达标率、自来水入户率。积极推进农网改造，重点解决自然村（组）低电压等供电质量问题，强化集中养殖区、设施农业区电力保障。按照“群众自愿、政府扶持、方便生产、永久定居”的要求，对生态环境极度恶化、生产生活条件差的区域，有计划、有组织地实施移民搬迁，建立移民片带，集中改善基础条件，培育后续致富产业，确保搬迁农民近期生活有保障、长远致富有门路。今年重点抓好55个整村推进、7个整乡推进项目建设，确保15万人稳定脱贫。

打好以“五变”为牵引的特色农业攻坚战。秉持高产、优质、高效、生态、安全的理念，坚持数量质量并重、增产增收并举，提高农业规模化经营、专业化生产水平。紧抓粮食生产不放松，推广双垄沟播面积300万亩以上，种植优质小杂粮100万亩，粮食总产量稳定在150万吨以上。在确保粮食安全的前提下，加快调整种植、养殖、农林布局结构，把肉羊、苹果作为富民增收的优势主导产业，把蔬菜、苗木作为脱贫致富的区域特色产业，因地制宜，分类推进。在北部山区，鼓励群众漫山遍野种草、家家户户养畜，引导粮农变牧民；在中南部原区，大力发展苹果产业，引导粮农变果农；在川台地，扶持发展设施蔬菜，引导粮农变菜农；在子午岭林缘区和宜林荒山区，大力发展苗林产业，引导粮农变林农；在城镇和园区，大力发展农产品精深加工业和优势资源开发配套服务业，引导农民变市民。全年新栽苹果10万亩，瓜菜面积稳定在100万亩以上。抓好现代畜牧业全产业链试点工作，促进养殖规模化发展，新建养殖场200个，肉牛、肉绒羊、生猪饲养量增速均达到20%以上。以土地流转和农民专业合作社为模式，以现代农业综合示范工程为载体，鼓励土地承包经营权向专业大户、家庭农场、农民合作社和农业企业流转，大力培育新型农业经营主体。今年建办农民合作社100个，新建和改扩建农产品加工销售企业20户以上，形成“建一个龙头、兴一项产业、富一方农民”的发展格局。

实施以培育新型农民为主的技能培训工程。针对农民缺技术、缺信息的问题，整合资源，因需施教，培养生产型、经营型、技能带动型、技术创新型和社会服务型“五型人才”。全年培训农民10万人（次），培训村干部、种养大户、合作社管理人员1000人，提升农村实用人才和村干部引领发展能力、种养大户带动致富能力、农民合作社综合服务能力。加快农业信息网络建设，帮助农民发展生产、开拓市场。

六、始终以人民为中心，着力保障和改善民生

坚持把促进社会公平正义、增进人民福祉作为政府工作的出发点和落脚点，不断提高公共服务能力和均等化水平，让人民群众共享改革发展成果。

实施创新驱动战略，建立产学研协同创新机制，提升企业技术创新能力。开展科技兴企、科技富民行动，完善科技项目成果和创新人才奖励机制，提高科技成果转化率和科技知识普及率。

统筹城乡教育资源配置，促进县域义务教育均衡发展。加快庆化实验学校、长庆中学和青少年课外综合实践基地建设，实施庆阳七中整体搬迁工程。推进“班班通”信息化工程建设，改善办学条件。加强学前教育、特殊教育和职业技术教育，筹建庆阳职业技术学院。

深入推进城乡文化一体化，开工建设传媒大厦、大剧院等重点文化工程，推进“三馆一站一屋”免费开放，实施农村电影公共服务等文化惠民工程。引导、鼓励社会力量发展公共文化事业，繁荣文化艺术创作。办好第十二届中国庆阳香包民俗文化节，组织参加省十三届运动会，承办好全省第八届民族运动会。

深化医疗卫生体制改革，建立健全统筹城乡的医疗保障模式和医疗保险管理体制。启动实施中医药综合改革试点示范、中医药养生保健旅游创新区建设。加快市妇女儿童医院和县、乡医疗卫生机构改扩建、村级卫生室标准化建设，全面建成市第二人民医院并投入使用；强化医疗卫生人才队伍建设，提升医疗服务能力。加强计划生育工作，完善利益导向机制，稳定低生育水平。

深入开展全民创业促进就业行动，完善创业政策和服务体系，鼓励更多的城乡劳动者通过自主创业实现就业。引导高校毕业生到基层、企业和社会组织就业。做好失业人员、复转军人、困难家庭就业帮扶工作。多渠道开发就业岗位，建成6个创业孵化基地，新增城镇就业4万人。

扩大“五项保险”覆盖面，提高社会保障能力。积极发展民政福利和残疾人、慈善事业，健全农村留守儿童、留守妇女、留守老年人关爱服务体系。创新社会救助机制和办法，构筑保障困难群众基本生活的防线。

加大保障性住房供给，加快棚户区和农村危房改造，有计划、有步骤、分层次地解决中低收入家庭住房困难。全年新建保障性住房1.46万套，其中棚户区改造1.3万套，力争改造农村危房（窑）1.5万户以上。

深化平安和谐庆阳建设，强化普法宣传教育，深入推进领导干部下访接访，让群众问题能反映、矛盾能化解、权益有保障。深入开展安全生产大排查、大整治，强化道路交通、消防、校园、产品质量监管。加快建立食品药品检测、监管、统筹、监督体系，确保人民群众“舌尖上的安全”。抓好“双拥”共建，加强国防后备力量建设。依法做好民族宗教工作，继续加大民族乡村扶贫攻坚力度。创新社会治理和公共服务，优化社区管理和服务功能，健全完善立体化社会治安防控体系，依法打击各类违法犯罪活动，保障人民安居乐业、社会安定有序。

在认真落实中央和省上惠民政策、完成省上确定的10项23件为民办实事项目的基础上，全力落实16件民生实事。（1）扶持1800名高校毕业生到非公经济组织就业。（2）为757个农村学校建设小伙房。（3）实施薄弱学校标准化建设及D级危房消除加固工程。（4）维修改造253个农村教学点。（5）免费开展婚前医学检查、农村妇女“两癌”预防普查。（6）为乡镇卫生院培训120名全科医生、60名产科医生。（7）对符合计划生育政策规定、年龄在50至59岁的农村夫妇，实施奖励扶助。（8）为全市8000名贫困残疾人配置康复器具。（9）扶持1200户贫困残疾人家庭改造危房（窑）。（10）新修通村公路1600公里。（11）解决农村17.82万人安全饮水问题。（12）在119个行政村开展“乡村舞台”工程建设试点，建办200个行政村民间文化社团。（13）在40个村开展“农家文化集市”建设试点。（14）开工建设市级养老基地。（15）开展城乡集贸市场衡器免费检定、诚信计量集贸市场体系建设。（16）新建、改造100所城乡幼儿园、100个小区蔬菜销售点、100个便民公厕、100个停车场，治理100条城市巷道。

七、加强自身建设，打造为民务实清廉政府

深入开展党的群众路线教育实践活动，把“为民”宗旨体现在执政理念上，把“务实”作风贯穿于工作实践中，把“清廉”要求落实在具体行动上，提高政府执行力和公信力。

解放思想，增强创新能力。把解放思想作为凝聚力量、促进创新、推动发展的强大武器，围绕中心聚焦发力，凝聚合力促进发展。用世界眼光、战略思维来审视发展和谋划工作，以全面深化改革的有力举措，锐意创新，主动作为，冲破思想观念的束缚，突破利益固化的藩篱，最大限度地汇聚加快富民兴市的内生动力，让一切劳动、知识、技术、管理、资本的活力竞相迸发，让一切创造社会财富的源泉充分涌流。

依法行政，建设法治政府。自觉接受人大法律监督和政协民主监督，认真办理人大代表议案、建议和政协委员提案。健全规范性文件备案审查制度和行政执法组织体系。完善重大决策规则和程序，强化决策审查、决策监督和决策责任。严格依法办事，促进法治政府、法治社会建设。强化政务公开，接受社会监督。加快综合应急信息平台建设，健全防灾减灾体系，提高防范和应对突发事件的能力。

改进作风，做到勤政廉政。严格执行中央“八项规定”、国务院“约法三章”和省、市委各项规定，强化监督检查，严格问责问效。带头说实话、办实事，防止急功近利、欺上瞒下；带头有令必行、有禁必止，防止推诿扯皮、政令不畅；带头深入实际、掌握实情，防止走马观花、形式主义；带头联系群众、贴近群众，防止高高在上、官僚主义；带头主动作为、激情干事，防止不思进取、享乐主义；带头勤俭节约、艰苦奋斗，防止铺张浪费、奢靡之风。严格遵守党的政治纪律、组织纪律、工作纪律、财经纪律和生活纪律，全面落实党风廉政建设责任制，加强政府工作人员教育管理，多打“预防针”，筑牢“防火墙”。整章建制、强化约束，严格用制度管人、管权、管事。加强对工程建设、土地管理、产权变动、公共资源交易、政府采购和资源开发等领域的监管和整治。严格审计监督、行政监察，深入开展专项治理，坚决纠正行业不正之风，确保干部清正、政府清廉、政治清明。

各位代表，人民对美好生活的向往，就是我们的奋斗目标。让我们在省委、省政府和市委的坚强领导下，在市人大、市政协的监督支持下，凝聚全市人民的智慧和力量，为圆满完成各项目标任务、加快全面建成小康社会、建设幸福美好新庆阳而努力奋斗！
